# More Retrieval Attempts are Associated with Poorer Functional Outcome After Unsuccessful Thrombectomy

**DOI:** 10.1007/s00062-021-01054-w

**Published:** 2021-07-08

**Authors:** F. Flottmann, N. van Horn, M. E. Maros, H. Leischner, M. Bechstein, L. Meyer, M. Sauer, M. Deb-Chatterji, A. Alegiani, G. Thomalla, J. Fiehler, C. Brekenfeld, GSR investigators

**Affiliations:** 1 Department of Diagnostic and Interventional Neuroradiology, University Medical Center Hamburg-Eppendorf, Martinistraße 52, 20246 Hamburg, Germany; 2 Department of Neuroradiology at the Center for Preventive Medicine and Digital Health, Medical Faculty Mannheim, Heidelberg University, Mannheim, Germany; 3 Department of Biomedical Informatics at the Center for Preventive Medicine and Digital Health, Medical Faculty Mannheim, Heidelberg University, Mannheim, Germany; 4 Department of Diagnostic and Interventional Radiology and Nuclear Medicine, University Medical Center Hamburg-Eppendorf, Hamburg, Germany; 5 Department of Neurology, University Medical Center Hamburg-Eppendorf, Hamburg, Germany

**Keywords:** Ischemic stroke, Thrombectomy, Endovascular therapy, Retrieval attempts, Prognostic factors

## Abstract

**Purpose:**

In mechanical thrombectomy, it has been hypothesized that multiple retrieval attempts might the improve reperfusion rate but not the clinical outcome. In order to assess a potential harmful effect of a mechanical thrombectomy on patient outcome, the number of retrieval attempts was analyzed. Only patients with a thrombolysis in cerebral infarction (TICI) score of 0 were reviewed to exclude the impact of eventual successful reperfusion on the mechanical hazardousness of repeated retrievals.

**Methods:**

In this study 6635 patients who underwent endovascular thrombectomy (EVT) for acute large vessel occlusion (LVO) from the prospectively administered multicenter German Stroke Registry were screened. Insufficient reperfusion was defined as no reperfusion (TICI score of 0), whereas a primary outcome was defined as functional independence (modified Rankin scale [mRS] 0–2 at day 90). Propensity score matching and multivariable logistic regressions were then performed to adjust for confounders.

**Results:**

A total of 377 patients (7.8%) had a final TICI score of 0 and were included in the study. After propensity score matching functional independence was found to be significantly more frequent in patients who underwent ≤ 2 retrieval attempts (14%), compared to patients with > 2 retrieval attempts (3.9%, OR 0.29, 95% CI 0.07–0.73, *p* = 0.009). After adjusting for age, sex, admission NIHSS score, and location of occlusion, more than two retrieval attempts remained significantly associated with lower odds of functional independence at 90 days (OR 0.2, 95% CI 0.07–0.52, *p* = 0.002).

**Conclusion:**

In patients with failure of reperfusion, more than two retrieval attempts were associated with a worse clinical outcome, therefore indicating a possible harmful effect of multiple retrieval attempts.

**Supplementary Information:**

The online version of this article (10.1007/s00062-021-01054-w) contains supplementary material, which is available to authorized users.

## Introduction

Endovascular thrombectomy (EVT) has been established as a standard practice in large-vessel occlusion (LVO) stroke patients [1]; however, often more than one retrieval attempt is needed in order to achieve complete reperfusion [2]. It has previously been described that reperfusion within the first retrieval attempt will lead to a better clinical outcome [2–5], especially if achieved by the first-pass retrieval [5–7]. One differentiating factor was that previous studies included all reperfusion grades; hence, presenting several confounders that are difficult to adjust for time of ischemia, which differs if reperfusion is achieved after first retrieval vs. multiple retrievals [8] and reperfusion grade [9] as well as reperfusion status after each retrieval (e.g. TICI 2b after the first two retrievals with final TICI 3 score after third attempt vs. TICI 0 after the first two retrievals and sudden TICI 3 reperfusion after third attempt) [10].

In the present study, we screened a multicenter stroke registry and selected patients who showed no reperfusion (TICI 0) in order to minimize the effect of the abovementioned confounders. We hypothesized that in this subset of patients higher numbers of retrieval attempts are associated with inferior clinical outcome.

## Material and Methods

### Patient Selection

A total of 6635 patients from the German Stroke Registry—Endovascular Treatment (GSR-ET 06/2015-12/2019; ClinicalTrials.gov Identifier: NCT03356392) were screened for inclusion. The GSR-ET is an ongoing, open-label, prospective, multicenter registry of consecutively recruited EVT patients, with 25 participating stroke centers in Germany [11].

The inclusion criteria for this study were: (1) acute large-vessel occlusion (LVO) stroke in patients > 18 years, (2) decision to perform EVT, (3) available data for admission National Institutes of Health Stroke Scale (NIHSS) score; administration of i.v. thrombolysis; number of retrieval attempts; final TICI score documented by DSA, and for the outcome to be assessed according to the modified Rankin scale (mRS) at 90 days (mRS90), (4) documented final TICI score of 0.

Study protocols and procedures were conducted in compliance with the Declaration of Helsinki and in accordance with ethical guidelines (the leading ethics committee of the Ludwig-Maximilians University Munich approved the GSR-ET, as well as the approval from the local ethics committees of the participating hospitals).

### Endovascular Thrombectomy

Endovascular thrombectomy was performed according to the clinical routine of the referring stroke center and in accordance with current guidelines [11, 12]. The technical approach was chosen by the attending neurointerventionalist who also made the decision when to terminate the procedure or to perform multiple retrieval attempts.

### Data Acquisition and Management

Data acquisition was performed according to the protocol of the GSR-ET, as has been previously described [11–13]. In summary, data were collected by local neurointerventionalists and neurologists and subsequently underwent standardized quality checks to control for consistency, plausibility, and integrity.

The final TICI score was assessed on the last DSA series by the attending interventionalist. The number of retrievals was documented by the neurointerventionalist immediately after the intervention and included aspiration attempts as well as retrievals with stent retriever devices. The mRS score was assessed at 90 days.

### Statistical Analyses

All analyses were performed with the R statistics program (v4.0.5, R Core Team 2021, Vienna, Austria; Rstudio IDE v. 1.4.1106, Boston, MA, USA). Normally distributed variables are displayed as mean and standard deviation (SD), and were compared with the Welch’s t‑test for unequal variances. Non-normally distributed data are displayed as median and interquartile range (IQR) and were compared with the Kruskal Wallis or Mann-Whitney-Wilcoxon tests. Categorical variables are reported as proportions and were compared by means of the χ^2^-test or Fisher’s exact test, when appropriate.

The primary outcome of interest was functional independence, defined by mRS at day 90 (mRS90) of 0–2 (see Table 1). The secondary outcome was the mRS90 as factor variable on an ordinal scale. The main explanatory variable of interest was the number of retrieval passes performed, which was dichotomized at the median number of retrieval attempts for all patients. In order to robustly estimate the effect of retrieval passes on mRS90 while controlling for key pretreatment patient characteristics, we performed propensity score matching (PSM) [14] using the MatchIt package [15] with a 1:1 ratio without replacement, using the nearest neighbor matching algorithm with a caliper width of 0.25, which is appropriate for estimating the average treatment effect in the treated population [16]. Propensity scores were calculated using the following covariates based on previous publications and clinical relevance: age, sex, prestroke mRS of > 1, NIHSS at admission, location of occlusion and administration of i.v. thrombolysis [5, 17]. The same covariates were used for fitting a generalized linear model with binomial link function (primary analysis) with functional independence as the dependent variable on the original cohort (Table 2). Likewise, as a secondary analysis, this same model formula was used in an ordinal logistic regression model with the proportional odds assumption [18] (Supplemental Table 1). As a sensitivity analysis, the binary logistic regression model was additionally tested on a subset of the initial (non-matched) collective that incorporated all available baseline variables with less than 10% missing values (Supplemental Table 2). *P*-values < 0.05 were considered statistically significant and were not adjusted for multiple testing due to the explanatory nature of this investigation.

**Table 1:** Comparison of clinical and treatment characteristics before and after propensity score matching^a^

	Before propensity score matching			After propensity score matching		
	≤ 2 retrievals *N* = 233	> 2 retrievals *N* = 144	*p*-value	≤ 2 retrievals *N* = 129	> 2 retrievals *N* = 129	*p*-value
*Age, years (mean, SD)*	76.5 (12.6)	73.8 (13)	**0.041**	74.5 (13.5)	74.5 (13.2)	0.978
*Female*	123 (52.8)	76 (52.8)	1	71 (55)	69 (55)	0.901
*Hypertension*	195 (84.1)	116 (81.1)	0.554	110 (85.3)	105 (81.4)	0.504
*Diabetes mellitus*	52 (22.5)	35 (24.6)	0.728	30 (23.3)	32 (24.8)	0.884
*Dyslipidemia*	77 (33.3)	56 (39.4)	0.279	44 (34.1)	53 (41.1)	0.304
*Atrial fibrillation*	101 (43.7)	54 (38.0)	0.329	50 (38.8)	50 (38.8)	1
*Smoking history*	–	–	0.276	–	–	0.860
*Current smoker*	29 (13.4)	21 (16.3)	–	17 (14.4)	18 (15.5)	–
*Non-smoker*	169 (77.9)	91 (70.5)	–	88 (74.6)	83 (71.6)	–
*Previous smoker*	19 (8.8)	17 (13.2)	–	13 (11)	15 (12.9)	–
*NIHSS on admission (median, Q1Q3)*	15 [9–19]	16 [10–20]	0.318	15 [10–18]	16 [10–20]	0.741
*Prestroke mRS score (median, Q1–Q3)*	0 [0–2]	0 [0–2]	0.265	1 [0–2]	0 [0–2]	0.433
*ASPECTS on admission (anterior circulation only, median, Q1–Q3)*^*b*^	8 [7–10]	8 [7–10]	0.324	8 [7–9]	8 [7–10]	0.904
*Left side occlusion*	117 (51.1)	71 (49.7)	0.804	67 (51.9)	68 (49.3)	0.465
*Location of vessel occlusion*						
*Tandem occlusion*	23 (10.3)	9 (6.3)	0.260	7 (5.4)	9 (7)	0.796
*ICA*	77 (34.4)	52 (36.4)	0.782	47 (36.4)	48 (37.2)	1
*M1 proximal*	64(28.6)	36 (25.2)	0.554	34 (26.4)	32 (24.8)	0.887
*M1 distal*	26 (11.6)	24 (16.8)	0.210	20 (15.5)	21 (16.3)	1
*M2*	55 (24.6)	27 (18.9)	0.253	30 (23.3)	26 (20.2)	0.651
*Posterior circulation*	26 (11.6)	12 (8.4)	0.418	6 (4.7)	10 (7.8)	0.439
*Intravenous tPA*	108 (46.4)	51 (35.4)	**0.048**	52 (40.3)	48 (37.2)	0.701
*Onset to admission, min (median, Q1–Q3)*^*c*^	149 [57–245]	137 [59–213]	0.436	149 [57–257]	138 [57–227]	0.390
*Stroke etiology*	–	–	0.123	–	–	0.887
*Cardioembolism*	108 (47.4)	63 (44.1)	–	56 (43.4)	59 (45.7)	–
*Dissection*	4 (1.8)	1 (0.7)	–	1 (0.8)	0 (0)	–
*Atherosclerosis*	73 (32.0)	36 (25.2)	–	37 (28.7)	36 (27.9)	–
*Other determined etiology*	12 (5.3)	9 (6.3)	–	10 (7.8)	9 (7)	–
*Unknown etiology*	31 (13.6)	33 (23.1)	–	25 (19.4)	25 (19.4)	–
*Onset to groin puncture, min (median, Q1–Q3)*	225 [151–320]	213 [156–270]	0.229	220 [158–323]	213 [157–279]	0.355
*Groin puncture to final TICI, min (median, Q1–Q3)*	52 [30–69]	97 [63–110]	**0.001**	63 [30–83]	85 [63–107]	0.063
*Onset to final TICI, min (median, Q1–Q3)*	255 [217–359]	328 [302–383]	0.144	310 [228–406]	346 [302–410]	0.443
*Dissection/perforation*	21 (9)	8 (5.6)	0.305	12 (9.3)	7 (5.4)	0.340
*sICH*	11 (4.8)	9 (6.3)	0.675	4 (3.1)	9 (7.1)	0.249
*NIHSS after 24* *h*	19 [9–23]	19 [14–25]	**0.012**	18 [9–24]	19 [14–25]	0.052
*NIHSS at discharge*	13 [6–18]	16 [11–31]	**<** **0.001**	14 [6–18]	16 [11–29]	**0.007**
*mRS score at 90 days (median, Q1–Q3)*	6 [4–6]	6 [4–6]	0.078	6 [4–6]	6 [4–6]	0.130
*Mortality*	123 (52.8)	83 (57.6)	0.417	69 (53.5)	74 (57.4)	0.616
*Good outcome (mRS90* *≤* *2)*	34 (14.6)	7 (4.9)	**0.005**	18 (14)	5 (3.9)	**0.009**

Bold *p*-values indicate statistical significance at the < 0.05 level

*NIHSS* National Institutes of Health Stroke Scale; *mRS* modified Rankin scale; *ASPECTS* Alberta Stroke Program Early Computed Tomography Score; *ICA* intracranial carotid artery; *M1* first segment of middle cerebral artery; *M2* second segment of middle cerebral artery; *tPA* tissue plasminogen activator; *TICI* thrombolysis in cerebral infarction; *sICH* symptomatic intracranial hemorrhage; *mRS* modified Rankin scale

^a^Data are *n* (%), unless otherwise indicated

^b^Available for 311/377 patients

^c^Available for 217/377 patients

**Table 2:** Summary table of predictor estimates of the binary logistic regression model with functional independence (mRS at day 90 of 0–2) as the dependent variable

Predictor variable	Coefficient^b^	SD	OR (95%CI)	*p*-value
*Age*^*a*^	−0.05	0.01	0.95 (0.92–0.97)	**<** **0.001**
*Female*	−0.55	0.41	0.57 (0.25–0.97)	0.172
*Pre-stroke mRS* *>* *1*	−054	0.61	0.59 (0.15–1.81)	0.384
*NIHSS on admission*^*a*^	−0.12	0.03	0.89 (0.83–0.95)	**<** **0.001**
*Tandem occlusion*	−1.48	1.31	0.23 (0.01–2.25)	0.260
*ICA*	−0.19	0.94	0.83 (0.12–4.71)	0.383
*M1 proximal*	−0.26	0.98	0.77 (0.10–4.76)	0.794
*M1 distal*	0.43	0.96	1.53 (0.21–9.40)	0.656
*M2*	0.25	0.91	1.28 (0.19–7.11)	0.785
*Posterior circulation*	0.63	1.05	1.87 (0.22–13.90)	0.551
*>* *2 retrieval attempts*	−1.60	0.52	0.20 (0.07–0.52)	**0.002**
*Intravenous tPA*	0.35	0.41	1.42 (0.63–3.19)	0.397

357/377 patients with complete data were included in the analysis

Bold *p*-values indicate statistical significance at the < 0.05 level

*mRS* modified Rankin scale; *NIHSS* National Institutes of Health Stroke Scale; *ICA* intracranial hemorrhage; *M1* first segment of middle cerebral artery; *M2* second segment of middle cerebral artery; *tPA* tissue plasminogen activator, SD, OR, CI

^a^Age and NIHSS were treated as continuous variables

^b^Coefficients are reported on the logit scale

## Results

Of 6635 patients screened for inclusion, 377 met the inclusion criteria (7.8%). Mean age was 75 (±12.8) years, median NIHSS score on admission was 15 (IQR 9–19), and median number of device passes was 2 (IQR 0–4). A good clinical outcome (mRS ≤ 2) was observed in 41 patients (10.9%) and the mortality rate was 54.6% (median mRS90 of 6).

Propensity score matching (PSM) was performed at the median of device passes over all included cases. Thus, cases with > 2 device passes (*n* = 129) were considered “treated” and paired with available controls receiving ≤ 2 device passes (*n* = 129) that had the closest propensity score to them. Unmatched control cases were excluded from univariate analyses (*n* = 119). Baseline data as well as clinical and procedural outcome measures for unmatched and matched patient groups are displayed in Table 1.

**Fig. 1:**
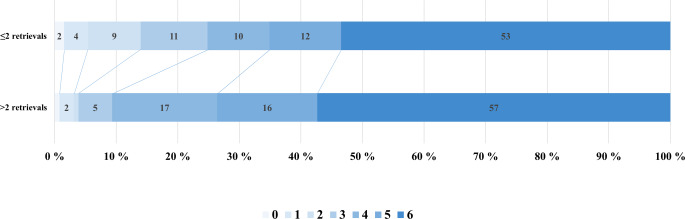
Ordinal modified Rankin scale after 90-day follow-up (mRS 90) in patients without reperfusion (TICI 0) after ≤ 2 vs. > 2 retrieval attempts (*n* = 258, 1:1 propensity-score matched patients). Functional independence (mRS 0–2) was observed in 18/129 patients (14%) with ≤ 2 retrieval attempts, and 5/129 patients (3.9%) with > 2 retrieval attempts

Before PSM age, time from groin puncture to final TICI and NIHSS at 24 h were significantly different between groups, while NIHSS at discharge remained significant (*p* = 0.007) throughout pair matching. Of 129 matched patients with ≤ 2 retrieval attempts, 18 (14%) had a good clinical outcome, compared to only 5 patients (3.9%) with > 2 retrieval attempts (Table 1, Fig. 1). This difference was highly significant in univariate analysis (OR 0.29, 95% CI 0.07–0.73, *p* = 0.009). There was no significant difference in the occurrence of symptomatic intracranial hemorrhage (sICH) between the two groups with 4 (3.1%) for ≤ 2 retrieval attempts compared to 9 (7.1%) for > 2 retrievals (*p* = 0.25).

In the multivariable binary logistic regression fitted on complete cases (*n* = 357, Table 2) of the original cohort using the same covariates as for the PSM, the primary outcome (mRS ≤ 2, functional independence) was less likely to be achieved in patients with > 2 retrievals (OR 0.2, 95% CI 0.07–0.52, *p* = 0.002) after adjusting for confounders. Furthermore, older age (OR 0.95, 95% CI 0.92–0.97, *p* < 0.001) and higher NIHSS scores on admission (OR 0.89, 95% CI 0.83–0.95, *p* < 0.001) were associated with lower odds of functional independence (Table 2).

Similarly, in the secondary analysis using multivariable ordinal logistic regression (Supplemental Table 1), a higher mRS90 was associated with > 2 retrievals (OR 1.68, 95% CI 1.08–2.64, *p* = 0.022). Furthermore, older age (OR 1.05, 95% CI 1.03–1.07, *p* < 0.001), a pre-stroke mRS of > 1 (OR 3.02, 95% CI 1.73–5.42, *p* < 0.001), and a higher NIHSS on admission (OR 1.10, 95% CI 1.07–1.14, *p* < 0.001) were associated with higher mRS scores on day 90.

As a sensitivity analysis, a multivariable binary logistic regression model was fitted including the complete cases (*n* = 319) of all baseline variables from Table 1 with less than 10% missing values (Supplemental Table 2). Again, functional independence was less likely in patients with > 2 retrievals (OR 0.15, 95% CI 0.04–0.48, *p* = 0.003). Older age per year (OR 0.95, 95% CI 0.91–0.98, *p* = 0.007) and higher NIHSS scores on admission (OR 0.87, 95% CI 0.80–0.94, *p* < 0.001) were associated with lower odds of functional independence. Atrial fibrillation narrowly missed the significance threshold for good a clinical outcome (OR 4.57, 95% CI 0.99–24.64, *p* = 0.062).

## Discussion

This study investigated the influence of the number of retrieval attempts performed during mechanical thrombectomy for acute ischemic stroke on clinical outcome in patients with no reperfusion (TICI score of 0). We found a significant association between more than two retrieval attempts and higher mRS scores at 90 days, even after propensity score matching and correcting for age, sex, pre-stroke mRS, NIHSS score on admission, location of occlusion and administration of i.v. thrombolysis.

Several studies have shown a decline in good clinical outcome if multiple retrieval attempts are performed and have reported best outcome results if reperfusion was achieved by just one retrieval, also known as the first-pass effect [2–7]; however, these studies included all reperfusion grades, making the analysis susceptible to bias. The number of retrievals is associated with increased procedure time [8] and patients with early successful reperfusion will have shorter ischemia times than patients with delayed reperfusion. Hence, it could be the procedure duration and thereby the time of brain ischemia as a latent variable that is responsible for an impaired clinical outcome, not the number of retrievals. Furthermore, these studies included TICI 2b and TICI 3 reperfusion grades were declared as “successful reperfusion”; however, substantial differences in clinical outcomes have been reported between TICI 2b and TICI 3 cases [9]. Furthermore, the situation is complicated by the fact that not only first-pass reperfusion but sudden reperfusion at later points in time (from TICI 0 to TICI 2b/3) is also associated with improved clinical outcome [10].

These confounders have been addressed by Nikoubashman et al. [7], who only included patients with TICI 3 reperfusion and verified their results in a matched-pair analysis. They suggested that indeed the number of device passes rather than the procedure time itself is associated with the clinical outcome; however, the authors stated that differences in patient characteristics cannot be ruled out completely.

To address these potential biases we exclusively included patients with a TICI score of 0 and performed 1:1 propensity score matching as well as detailed multivariable analyses of patients with more than two retrieval attempts, adjusted for typical confounders. We could thereby confirm the results of the previous retrospective studies that the number of retrieval attempts is independently associated with a worse clinical outcome. As Nikoubashman et al. stated [7] there are two possible explanations for this phenomenon:

First, the first-pass effect could be real and the number of retrievals increases the risk of vessel injury and subsequent symptomatic intracranial hemorrhage (sICH) [19–21]; however, we did not observe a statistically significant increased number of sICH in our study, although this comparison might lack statistical power due to the small number of patients in our cohort. Furthermore, each retrieval attempt is associated with risk of dispersing small distal emboli, which could lead to impaired clinical outcome [22, 23]. Other possible reasons include the prolonged procedure time, the need for induction of general anesthesia and increased amounts of contrast agents [24, 25].

Second, the first-pass effect could be an epiphenomenon and the number of retrieval attempts merely the byproduct of other causes of impaired clinical outcome, e.g. for tandem occlusions of the cervical ICA and the M1 segment more retrieval attempts are performed compared to single M1 occlusions. Therefore, we adjusted for the location of occlusion in our multivariable analysis. Furthermore, the clot histology has been associated with reperfusion results, higher number of retrievals and thrombus fragmentation which might impair the clinical condition [26]. We could not correct for clot histology or composition in our analyses as it is not part of the registry.

Another important point is the phenomenon of spontaneous reperfusion which is associated with improved outcome [27]. It has been reported that in the first 24 h from symptom onset, 24% of untreated patients and 46% of patients with iv-rtPA achieve successful reperfusion [28]. Accordingly, some of the patients with TICI 0 at the end of EVT might be reperfused later on. A hypothesis to explain our findings is that multiple unsuccessful retrieval attempts diminish the chance of subsequent spontaneous reperfusion due to vessel wall damage or mechanical alteration of the occluding thrombus. This could be tested by performing vessel imaging in a cohort of TICI 0 patients on day 1 after EVT, to assess the reperfusion rate depending on the number of retrievals performed; however, these data are not included in the GSR-ET.

The most important bias in retrospective trials assessing the number of retrieval attempts remains true for the present study: the reasons for performing multiple attempts vs. terminating a procedure are heterogeneous, for example one might perform multiple attempts in a young patient with large penumbral tissue as opposed to an older patient with small penumbral tissue. Furthermore, the reasons for failure of reperfusion and therewith inclusion in the present study are heterogeneous and include difficulties in accessing the intracranial vessels, passing the thrombus/occlusion, and mobilizing/removing the thrombus as well as the event of reocclusions [13, 29, 30]. Due to the study design, there was no information on the technical approach of EVT (e.g. aspiration or stent-retriever as first-line approach) available.

The results of the present study support the hypothesis that extended endovascular intervention with increased number of retrieval attempts leads to a poorer clinical outcome. This might impact treatment decision making, if after several retrieval attempts, late successful reperfusion also does not result in an improved outcome [5]. To define the optimal number of retrieval attempts is beyond the scope of this study and would require a randomized controlled trial to correct for the abovementioned biases.

## Conclusion

In patients with failure of reperfusion, more than two retrieval attempts were independently associated with a worse clinical outcome after adjusting for age, sex, admission NIHSS score, and the site of occlusion.

## Supplementary Information

Supplemental Tables: Supplemental 1: Multivariable ordinal regression analysis with mRS 90 as outcome variable. Supplemental 2: Sensitivity analysis including all baseline variables with predictor estimates of the binary logistic regression model with functional independence (mRS at day 90of 0-2) as the dependent variable.

## References

[1] Turc G, Bhogal P, Fischer U, Khatri P, Lobotesis K, Mazighi M, Schellinger PD, Toni D, de Vries J, White P, Fiehler J (2019). European Stroke Organisation (ESO) – European Society for Minimally Invasive Neurological Therapy (ESMINT) guidelines on mechanical thrombectomy in acute ischemic stroke. J Neurointerv Surg.

[2] Flottmann F, Leischner H, Broocks G, Nawabi J, Bernhardt M, Faizy TD, Deb-Chatterji M, Thomalla G, Fiehler J, Brekenfeld C (2018). Recanalization rate per retrieval attempt in mechanical thrombectomy for acute ischemic stroke. Stroke.

[3] Seker F, Pfaff J, Wolf M, Ringleb PA, Nagel S, Schönenberger S, Herweh C, Möhlenbruch MA, Bendszus M, Pham M (2017). Correlation of thrombectomy maneuver count with recanalization success and clinical outcome in patients with ischemic stroke. AJNR Am J Neuroradiol.

[4] García-Tornel Á, Requena M, Rubiera M, Muchada M, Pagola J, Rodriguez-Luna D, Deck M, Juega J, Rodríguez-Villatoro N, Boned S, Olivé-Gadea M, Tomasello A, Hernández D, Molina CA, Ribo M (2019). When to stop. Stroke.

[5] Flottmann F, Brekenfeld C, Broocks G, Leischner H, McDonough R, Faizy TD, Deb-Chatterji M, Alegiani A, Thomalla G, Mpotsaris A, Nolte CH, Fiehler J, Maros ME (2021). Good clinical outcome decreases with number of retrieval attempts in stroke thrombectomy: beyond the first-pass effect. Stroke.

[6] Zaidat OO, Castonguay AC, Linfante I, Gupta R, Martin CO, Holloway WE, Mueller-Kronast N, English JD, Dabus G, Malisch TW, Marden FA, Bozorgchami H, Xavier A, Rai AT, Froehler MT, Badruddin A, Nguyen TN, Taqi MA, Abraham MG, Yoo AJ, Janardhan V, Shaltoni H, Novakovic R, Abou-Chebl A, Chen PR, Britz GW, Sun C-HJ, Bansal V, Kaushal R, Nanda A, Nogueira RG (2018). First pass effect: a new measure for stroke thrombectomy devices. Stroke.

[7] Nikoubashman O, Dekeyzer S, Riabikin A, Keulers A, Reich A, Mpotsaris A, Wiesmann M (2019). True first-pass effect. Stroke.

[8] Settecase F, McCoy DB, Darflinger R, Alexander MD, Cooke DL, Dowd CF, Hetts SW, Higashida RT, Halbach VV, Amans MR (2018). Improving mechanical thrombectomy time metrics in the angiography suite: stroke cart, parallel workflows, and conscious sedation. Interv Neuroradiol.

[9] Kleine JF, Wunderlich S, Zimmer C, Kaesmacher J (2017). Time to redefine success? TICI 3 versus TICI 2b recanalization in middle cerebral artery occlusion treated with thrombectomy. J Neurointerv Surg.

[10] García-Tornel Á, Rubiera M, Requena M, Muchada M, Pagola J, Rodriguez-Luna D, Deck M, Juega J, Rodríguez-Villatoro N, Boned S, Olivé-Gadea M, Tomasello A, Piñana C, Hernández D, Molina CA, Ribo M (2020). Sudden recanalization. Stroke.

[11] Alegiani AC, Dorn F, Herzberg M, Wollenweber FA, Kellert L, Siebert E, Nolte CH, von Rennenberg R, Hattingen E, Petzold GC, Bode FJ, Pfeilschifter W, Schäfer JH, Wagner M, Röther J, Eckert B, Kraft P, Pham M, Boeckh-Behrens T, Wunderlich S, Bernkopf K, Reich A, Wiesmann M, Mpotsaris A, Psychogios M, Liman J, Maier I, Berrouschot J, Bormann A, Limmroth V, Spreer J, Petersen M, Krause L, Lowens S, Kraemer C, Zweynert S, Lange KS, Thonke S, Kastrup A, Papanagiotou P, Alber B, Braun M, Fiehler J, Gerloff C, Dichgans M, Thomalla G (2019). Systematic evaluation of stroke thrombectomy in clinical practice: the German Stroke Registry endovascular treatment. Int J Stroke.

[12] Wollenweber FA, Tiedt S, Alegiani A, Alber B, Bangard C, Berrouschot J, Bode FJ, Boeckh-Behrens T, Bohner G, Bormann A, Braun M, Dorn F, Eckert B, Flottmann F, Hamann GF, Henn K-H, Herzberg M, Kastrup A, Kellert L, Kraemer C, Krause L, Lehm M, Liman J, Lowens S, Mpotsaris A, Papanagiotou P, Petersen M, Petzold GC, Pfeilschifter W, Psychogios M-N, Reich A, von Rennenberg R, Röther J, Schäfer J-H, Siebert E, Siedow A, Solymosi L, Thonke S, Wagner M, Wunderlich S, Zweynert S, Nolte CH, Gerloff C, Thomalla G, Dichgans M, Fiehler J (2019). Functional outcome following stroke thrombectomy in clinical practice. Stroke.

[13] Flottmann F, Broocks G, Faizy TD, McDonough R, Watermann L, Deb-Chatterji M, Thomalla G, Herzberg M, Nolte CH, Fiehler J, Leischner H, Brekenfeld C, GSR investigators (2020). Factors associated with failure of reperfusion in endovascular therapy for acute ischemic stroke: a multicenter analysis. Clin Neuroradiol.

[14] Brookhart MA, Schneeweiss S, Rothman KJ, Glynn RJ, Avorn J, Stürmer T (2006). Variable selection for propensity score models. Am J Epidemiol.

[15] Ho D, Imai K, King G, Stuart EA (2011). MatchIt: nonparametric preprocessing for parametric causal inference. J Stat Softw.

[16] McDonald JS, Brinjikji W, Rabinstein AA, Cloft HJ, Lanzino G, Kallmes DF (2015). Conscious sedation versus general anaesthesia during mechanical thrombectomy for stroke: a propensity score analysis. J Neurointerv Surg.

[17] Goyal M, Menon BK, van Zwam WH, Dippel DWJ, Mitchell PJ, Demchuk AM, Dávalos A, Majoie CBLM, van der Lugt A, de Miquel MA, Donnan GA, Roos YBWEM, Bonafe A, Jahan R, Diener H-C, van den Berg LA, Levy EI, Berkhemer OA, Pereira VM, Rempel J, Millán M, Davis SM, Roy D, Thornton J, Román LS, Ribó M, Beumer D, Stouch B, Brown S, Campbell BCV, van Oostenbrugge RJ, Saver JL, Hill MD, Jovin TG, HERMES collaborators (2016). Endovascular thrombectomy after large-vessel ischaemic stroke: a meta-analysis of individual patient data from five randomised trials. Lancet.

[18] Bath PMW, Lees KR, Schellinger PD, Altman H, Bland M, Hogg C, Howard G, Saver JL (2012). Statistical analysis of the primary outcome in acute stroke trials. Stroke.

[19] Bai Y, Pu J, Wang H, Yang D, Hao Y, Xu H, Zhang M, Geng Y, Wan Y, Wang W, Zhang H, Zi W, Liu X, Xu G (2018). Impact of retriever passes on efficacy and safety outcomes of acute ischemic stroke treated with mechanical thrombectomy. Cardiovasc Intervent Radiol.

[20] Bourcier R, Saleme S, Labreuche J, Mazighi M, Fahed R, Blanc R, Gory B, Kyheng M, Marnat G, Bracard S, Desal H, Consoli A, Piotin M, Lapergue B, ASTER Trial Investigators (2019). More than three passes of stent retriever is an independent predictor of parenchymal hematoma in acute ischemic stroke. J Neurointerv Surg.

[21] Maros ME, Brekenfeld C, Broocks G, Leischner H, McDonough R, Deb-Chatterji M, Alegiani A, Thomalla G, Fiehler J, Flottmann F (2021). Number of retrieval attempts rather than procedure time is associated with risk of symptomatic intracranial hemorrhage. Stroke.

[22] Chueh J-Y, Puri AS, Wakhloo AK, Gounis MJ (2016). Risk of distal embolization with stent retriever thrombectomy and ADAPT. J Neurointerv Surg.

[23] Schönfeld MH, Kabiri R, Kniep HC, Meyer L, Sedlacik J, Ernst M, Broocks G, Faizy TD, Cheng B, Thomalla G, Fiehler J, Hanning U (2020). Sub-angiographic peripheral emboli in high resolution DWI after endovascular recanalization. J Neurol.

[24] Brinjikji W, Pasternak J, Murad MH, Cloft HJ, Welch TL, Kallmes DF, Rabinstein AA (2017). Anesthesia-related outcomes for endovascular stroke revascularization. Stroke.

[25] Whitney E, Khan YR, Alastra A, Schiraldi M, Siddiqi J (2020). Contrast extravasation post thrombectomy in patients with acute cerebral stroke: a review and recommendations for future studies. Cureus.

[26] Goebel J, Gaida B-J, Wanke I, Kleinschnitz C, Koehrmann M, Forsting M, Moenninghoff C, Radbruch A, Junker A (2020). Is histologic thrombus composition in acute stroke linked to stroke etiology or to interventional parameters?. AJNR Am J Neuroradiol.

[27] Barber PA, Davis SM, Infeld B, Baird AE, Donnan GA, Jolley D, Lichtenstein M (1998). Spontaneous reperfusion after ischemic stroke is associated with improved outcome. Stroke.

[28] Rha J-H, Saver JL (2007). The impact of recanalization on ischemic stroke outcome: a meta-analysis. Stroke.

[29] Leischner H, Flottmann F, Hanning U, Broocks G, Faizy TD, Deb-Chatterji M, Bernhardt M, Brekenfeld C, Buhk J-H, Gellissen S, Thomalla G, Gerloff C, Fiehler J (2019). Reasons for failed endovascular recanalization attempts in stroke patients. J Neurointerv Surg.

[30] Kaesmacher J, Gralla J, Mosimann PJ, Zibold F, Heldner MR, Piechowiak E, Dobrocky T, Arnold M, Fischer U, Mordasini P (2018). Reasons for reperfusion failures in stent-retriever-based thrombectomy: registry analysis and proposal of a classification system. AJNR Am J Neuroradiol.

