# Supplementary material for: More Retrieval Attempts are Associated with Poorer Functional Outcome After Unsuccessful Thrombectomy
Source: Clin Neuroradiol. 2021 Jul 8;32(2):361–8. doi: 10.1007/s00062-021-01054-w (PMC9187527; doi:10.1007/s00062-021-01054-w)
Supplement: Supplementary file 1 — Supplemental Tables: Supplemental 1: Multivariable ordinal regression analysis with mRS 90 as outcome variable. Supplemental 2: Sensitivity analysis including all baseline variables with predictor estimates of the binary logistic regression model with functional independence (mRS at day 90of 0-2) as the dependent variable. [file 62_2021_1054_MOESM1_ESM.docx]

**Supplemental material**

| Supplemental Table 1: Multivariable ordinal regression analysis with mRS 90 as outcome variable. | | | | |
| --- | --- | --- | --- | --- |
|  | | | | |
| Predictor variable | **Coefficient^#^** | **SD** | **OR (95%CI)** | **p-value** |
| Age^a^ | 0.05 | 0.01 | 1.05 (1.03-1.07) | **<0.001** |
| Female | 0.29 | 0.22 | 1.34 (0.87-2.06) | 0.190 |
| Pre-stroke mRS | 1.11 | 0.29 | 3.02 (1.73-5.42) | **<0.001** |
| NIHSS on admission | 0.10 | 0.17 | 1.10 (1.07-1.14) | **<0.001** |
| Tandem occlusion | -0.13 | 0.47 | 0.88 (0.35-2.25) | 0.790 |
| ICA | 0.08 | 0.43 | 1.09 (0.47-2.57) | 0.848 |
| M1 proximal | -0.12 | 0.42 | 0.88 (0.39-2.04) | 0.772 |
| M1 distal | -0.58 | 0.47 | 0.56 (0.22-1.40) | 0.212 |
| M2 | -0.72 | 0.44 | 0.49 (0.21-1.15) | 0.010 |
| Posterior circulation | -0.51 | 0.56 | 0.60 (0.20-1.82) | 0.366 |
| Intravenous tPA | -0.32 | 0.22 | 0.73 (0.47-1.12) | 0.150 |
| >2 retrieval attempts | 0.52 | 0.23 | 1.68 (1.08-2.64) | 0.022 |
| Intercept - mRS level 0 to 1 | 0.70 | 0.80 |  | 0.386 |
| Intercept - mRS level 1 to 2 | 1.72 | 0.76 |  | **0.024** |
| Intercept - mRS level 2 to 3 | 2.42 | 0.76 |  | **0.001** |
| Intercept - mRS level 3 to 4 | 3.28 | 0.77 |  | **<0.001** |
| Intercept - mRS level 4 to 5 | 4.26 | 0.79 |  | **<0.001** |
| Intercept - mRS level 5 to 6 | 5.13 | 0.80 |  | **<0.001** |
| ^a^Age and NIHSS were treated as continuous variables; ^#^Coefficients are reported on the logit scale.  mRS – modified Rankin scale; NIHSS – National Institutes of Health Stroke Scale; ICA – Intracranial carotid artery; M1 – first segment of middle cerebral artery; M2 – second segment of middle cerebral artery; tPA – tissue plasminogen activator. 357/377 patients with complete data were included in the analysis.  Bold p-values indicate statistical significance at the <0.05 level. | | | | |

| Supplemental Table 2: Sensitivity analysis including all baseline variables* of the binary logistic regression model with functional independence (mRS at day 90 of 0-2) as the dependent variable. | | | | |
| --- | --- | --- | --- | --- |
|  | | | | |
| Predictor variable* | **Coefficient^#^** | **SD** | **OR (95%CI)** | **p-value** |
| Age^a^ | -0.52 | 0.02 | 0.95 (0.91-0.98) | **0.008** |
| Female | -0.80 | 0.53 | 0.45 (0.16-1.25) | 0.129 |
| Hypertension | -0.85 | 0.58 | 0.43 (0.14-1.36) | 0.143 |
| Diabetes mellitus | -1.10 | 0.74 | 0.33 (0.06-1.28) | 0.140 |
| Dyslipidemia | 0.24 | 0.52 | 1.27 (0.45-3.51) | 0.693 |
| Atrial fibrillation | 1.52 | 0.82 | 4.57 (0.99-24.64) | 0.062 |
| Non smoker | -0.51 | 0.62 | 0.60 (0.18-2.09) | 0.412 |
| Previous smoker | -0.42 | 0.86 | 0.65 (0.11-3.34) | 0.620 |
| NIHSS on admission^a^ | -0.14 | 0.04 | 0.87 (0.80-0.94) | **0.001** |
| Pre-stroke mRS score | -0.17 | 0.67 | 0.84 (0.20-3.00) | 0.799 |
| Left side occlusion | 0.77 | 0.52 | 2.15 (0.79-6.22) | 0.142 |
| Tandem occlusion | -2.12 | 1.37 | 0.12 (0.00-1.37) | 0.121 |
| ICA | -0.35 | 0.98 | 0.71 (0.09-4.60) | 0.726 |
| M1 proximal | -0.22 | 1.00 | 0.80 (0.10-5.42) | 0.824 |
| M1 distal | 0.64 | 1.03 | 1.90 (0.23-13.57) | 0.532 |
| M2 | 0.29 | 0.93 | 1.34 (0.20-7.93) | 0.751 |
| Posterior circulation | 0.11 | 1.21 | 1.11 (0.09-11.26) | 0.929 |
| Intravenous tPA | 0.05 | 0.48 | 1.05 (0.40-2.71) | 0.918 |
| Cardioembolism | -1.00 | 1.06 | 0.37 (0.05-3.13) | 0.344 |
| Atherosclerosis | 0.70 | 0.82 | 2.02 (0.44-11.58) | 0.392 |
| Other determined etiology | 0.33 | 1.15 | 1.38 (0.13-13.03) | 0.777 |
| >2 retrieval attempts | -1.89 | 0.64 | 0.15 (0.04-0.48) | **0.003** |
| ^a^Age and NIHSS were treated as continuous variables; ^#^Coefficients are reported on the logit scale.  NIHSS – National Institutes of Health Stroke Scale; mRS – modified Rankin scale; ICA – intracranial hemorrhage; M1 – first segment of middle cerebral artery; M2 – second segment of middle cerebral artery; tPA – tissue plasminogen activator.  *All baseline variables from Table 1 were included in the analysis, except those variables that had more than 10 % missing values (ASPECTS on admission, time from symptom onset to admission). 319/377 patients with complete data were included in the analysis.  Bold p-values indicate statistical significance at the <0.05 level. | | | | |
